# A New Ordered Transformation‐Induced Plasticity Enables Exceptional Strength and Ductility in a B2 Medium‐Entropy Alloy

**DOI:** 10.1002/advs.77739

**Published:** 2026-09-16

**Authors:** Chaohua Li, Yidong Wu, Boyuan Zheng, Xu Zhang, Xiongjun Liu, Wenli Song, Ning An, Chengbo Xiao, Xidong Hui

**Affiliations:** ^1^ State Key Laboratory for Advanced Metals and Materials University of Science and Technology Beijing Beijing China; ^2^ Institute of High Energy Physics Chinese Academy of Sciences Beijing China; ^3^ Spallation Neutron Source Science Center Dongguan China; ^4^ Institute of Materials Research Beijing Beiye Advanced Materials Co., Ltd Beijing China; ^5^ Science and Technology on Advanced High Temperature Structural Materials Laboratory Beijing Institute of Aeronautical Materials Beijing China

**Keywords:** B2 medium‐entropy alloy, intrinsic brittleness, metastability engineering, ordered martensitic transformation, strength‐ductility synergy

## Abstract

Ordered aluminide intermetallics are promising lightweight structural materials but suffer from limited room‐temperature tensile ductility due to the constrained dislocation activity inherent to their ordered lattices. Here, we demonstrate that controlled reduction of phase stability can overcome this limitation in B2‐ordered Ti‐Zr‐V‐Al medium‐entropy intermetallics. By tailoring the Al content to lower the stability of the B2 matrix while preserving long‐range order, the metastable alloy activates an ordered transformation‐induced plasticity (ordered TRIP) effect under tensile loading. This involves the cooperative operation of {110}<111> slip and an unprecedented stress‐induced B2 to D0_19_‐α_2_ martensitic transformation. The transformation follows an orientation relationship of {0001}α_2_//{110}B2 and <11‐20>α_2_//<100>B2, representing an atypical pathway distinct from conventional B2→B19′ or B2→B33 transformations. The designed alloy achieves a tensile elongation of about 7%, an ultimate tensile strength of about 1.1 GPa, and a specific yield strength of about 197 MPa·g^−1^·cm^3^, a combination of strength, ductility, and density that surpasses most reported lightweight B2 intermetallics. By establishing a controlled metastability strategy that couples dislocation plasticity with a novel ordered martensitic transformation, this work provides a new paradigm for designing ductile, high‐strength ordered alloys and may inspire similar approaches in other brittle ordered alloy systems.

## Graphical abstract

A novel B2‐ordered Ti‐Zr‐V‐Al medium‐entropy intermetallic is developed through controlled metastability engineering, which activates an ordered TRIP effect via a new B2→D0_19_‐α_2_ transformation with a distinctive {0001}α_2_//{110}B2, <11‐20>α_2_//<100>B2 orientation relationship. This alloy achieves an elongation of ∼7%, a strength of ∼1.1 GPa, and superior specific strength that surpasses most reported lightweight B2 intermetallics.

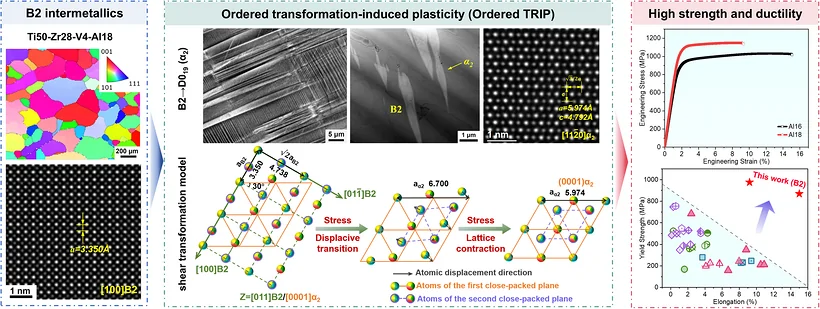

## 1 Introduction

The development of lightweight structural materials that combine high strength with appreciable tensile ductility at room temperature remains a long‐standing goal in physical metallurgy. Aluminide intermetallics (X‐Al, where X = Ni, Fe, Ti, etc.) are attractive candidates due to their low density, high specific strength, and good oxidation/corrosion resistance [1, 2, 3, 4, 5, 6]. However, their broader application is severely limited by poor room‐temperature tensile ductility, a direct consequence of the highly ordered atomic arrangement that restricts dislocation activity [2, 7, 8]. In contrast to disordered solid solutions, ordered intermetallics are governed by strict sublattice occupancy and near‐stoichiometric compositions, resulting in limited operative slip systems and high antiphase boundary (APB) energy, and a strong propensity for brittle cleavage or intergranular fracture [9, 10, 11].

The B2 structure, owing to its simple CsCl‐type lattice and relatively high symmetry, has long been considered a promising model system. In principle, it can support multiple slip modes, including {110}<111> and {001}<100>, suggesting the possibility of appreciable plastic deformability. Nevertheless, most binary B2 alloys (e.g., NiAl, FeAl) exhibit very limited tensile elongation (EL) at room temperature, typically failing by cleavage or intergranular fracture [5, 10, 12, 13, 14]. A key reason is that the high APB energy in ordered B2 lattices (e.g., ∼810 mJ/m^2^ for NiAl) strongly constrains the motion of <111> superdislocations, leaving deformation to be carried predominantly by the less effective <001> systems [1, 2]. This insufficient slip activity cannot accommodate uniform polycrystalline plastic deformation, leading to stress concentration at grain boundaries (GBs) and premature crack initiation.

Considerable efforts have therefore been devoted to alleviating the brittleness of B2 intermetallics, including reducing APB energy via alloying, introducing localized disorder near GBs, adjusting stoichiometry to generate anti‐site defects, and grain refinement [6, 15, 16, 17, 18, 19]. More recently, the incorporation of multiple principal elements into long‐range ordered lattices has been explored to alter lattice distortion and dislocation core structure [7, 20, 21, 22, 23, 24, 25]. However, these strategies often achieve only modest improvement in tensile ductility; excessive disorder tends to compromise the strengthening effect of long‐range order. Even when compressive deformability is enhanced, room‐temperature tensile plasticity of B2‐ordered alloys remains limited [24, 26, 27, 28, 29, 30, 31]. This suggests that modifying local order or chemistry alone is not sufficient, and that the deformation mode of the B2 lattice itself must be reconsidered.

Notably, metastability engineering has been extensively applied to medium– and high‐entropy alloys, in which controlled phase stability and transformation pathways have enabled outstanding strength‐ductility synergy [32, 33, 34, 35]. A few B2‐ordered alloys also exploit this approach via stress‐induced martensitic transformation (SIMT) to attain exceptional tensile ductility. The most well‐known examples are NiTi (B2→B19′) and ZrCoNi (B2→B33), which can achieve excellent tensile elongations at the cost of relatively low yield strengths (YS < 420 MPa) [36, 37, 38]. In these systems, the metastable B2 parent phase undergoes a diffusionless, displacive transformation to a martensitic phase under stress, generating transformation‑induced plasticity (TRIP). This TRIP effect effectively relaxes local stress concentrations, delays necking, and sustains work hardening. However, in conventional TRIP‑enabled B2 alloys, the transformation products are either monoclinic (B19′) or orthorhombic (B33) martensite, and the YS remains intrinsically low. Moreover, the application of this concept to other B2 aluminide intermetallics, which are of greater interest for lightweight structural applications, has not been successful, largely because the phase stability of Al‐containing B2 compounds is typically too high to allow SIMT under tensile stress.

Here, we propose a metastability engineering approach to deliberately lower the stability of a B2‐ordered Ti‐Zr‐V‐Al multicomponent aluminide, such that stress‐induced transformation to an ordered martensite becomes possible without sacrificing long‐range order strengthening. By tuning the Al content, we achieve a metastable single‐phase B2 matrix that co‑activates two deformation mechanisms under tensile loading: (i) {110}<111> dislocation slip, facilitated by a drastically reduced APB energy (∼38 mJ/m^2^), and (ii) a previously unreported stress‐induced B2→D0_19_‐α_2_ martensitic transformation. This transformation follows an orientation relationship of {0001}α_2_//{110}B2 and <11‐20>α_2_//<100>B2, an atypical pathway distinct from both the classical Burgers relationship in Ti alloys and the B2→B19′/B33 transformations known in NiTi and ZrCoNi alloys. We term this new deformation mechanism “ordered TRIP,” emphasizing that both the parent and the product phases are chemically ordered, and the transformation contributes to plasticity without disrupting long‐range order. The designed alloy achieves a tensile EL of ∼7%, an ultimate tensile strength (UTS) above 1.1 GPa, and a specific yield strength of ∼197  MPa·g^−1^·cm^3^, representing a combination of strength and ductility that surpasses most reported lightweight B2 intermetallics. By demonstrating that controlled metastability can unlock an ordered TRIP effect in a B2 aluminide, this work provides a new paradigm for overcoming the strength‑ductility trade‑off in ordered intermetallics. More broadly, the strategy may be extended to other brittle ordered alloy systems (such as L1_2_, L1_0_, and D0_19_ compounds) to design ductile, high‐strength lightweight structural materials.

## 2 Results and Discussion

### 2.1 Metastable B2 Ordered Matrix

**FIGURE 1:**
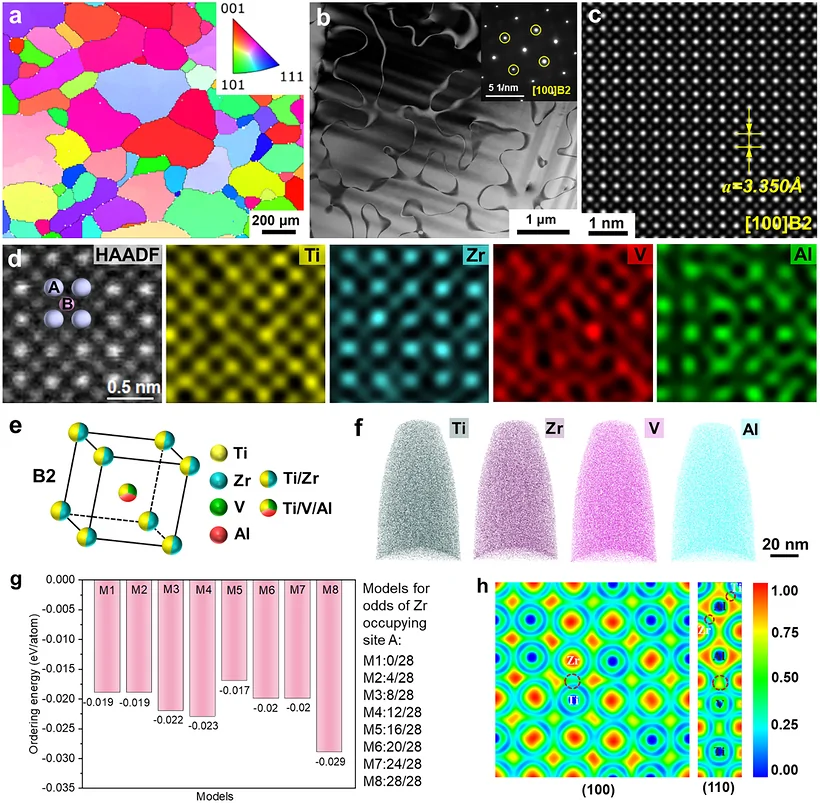
Microstructural morphology and B2 structure in the Al18 alloy. (a) Orientation map of polycrystalline morphology characterized by EBSD. (b) TEM bright‐field image of the as‐cast alloy. (c) B2 superlattice structure taken at high‐resolution HAADF‐STEM. (d) Atomic‐resolution HAADF‐STEM image and EDX mapping taken with the [100] zone axis, indicating the atomic occupations. Sites A and B represent the vertices and body‐centered positions, respectively. (e) The constructed model of the B2 structure. Ti and Zr atoms preferentially occupy the vertices, and the remaining atoms, Ti, V, and Al, occupy the body‐centered position. (f) Distribution of constituent elements without clustering. (g) The ordering energy of different unit cell models for the odds of Zr occupying site A. (h) ELF for (100) and (110) planes.

The metastability engineering was implemented in two alloys with nominal compositions Ti_50_Zr_46‐x_V_4_Al_x_ (at.%; hereinafter denoted Alx alloy; x = 16, 18). Electron backscatter diffraction (EBSD) shows a polycrystalline morphology with an average grain size of about 250 µm in both alloys (Figure 1a and Figure S1). X‐ray diffraction (XRD) and bright‐field transmission electron microscopy (TEM) confirm a single‐phase B2 structure (Figure 1b). Selected‐area electron diffraction (SAED) along the [100] zone axis shows characteristic superlattice reflections, and well‐defined antiphase domains (APDs) are visible, confirming long‐range chemical order. High‐resolution energy‐dispersive x‐ray spectroscopy (EDX) mapping combined with high‐angle annular dark‐field scanning TEM (HAADF‐STEM) reveals no detectable compositional difference between APDs and the matrix, indicating the chemical uniformity of the parent phase (Figure S2). Aberration‐corrected HAADF‐STEM determines the lattice parameters of 3.350 Å for Al18 and 3.384 Å for Al16 (Figure 1c and Figure S3a,b). Atomic‐scale HAADF‐STEM imaging with EDX established the specific site occupancy: Zr and Ti preferentially occupy the cube vertices (A site), while Ti, Al, and V are located at the body‐center positions (B site) (Figure 1d,e). Atom probe tomography (APT) confirms homogeneous elemental distribution without clustering (Figure 1f). The most stable configuration (Model 8) occurs when all Zr atoms occupy the vertex (Figure 1g), consistent with atomic size and electronegativity arguments: smaller, more electronegative V (electronegativity 1.63, radius 1.34 Å) and Al (1.61, 1.43 Å) favor body‐centered sites, while larger, less electronegative Ti (1.54, 1.47 Å) and Zr (1.33, 1.60 Å) favor the vertices, with the excess Ti accommodated at the body‐centered sites. The strong chemical affinity between Zr and Al further stabilizes the ordered configuration. These medium‐entropy alloy phase prediction parameters (Table S1) further elucidate stability trends, with mixing enthalpy and size mismatch both favoring the formation of intermetallics. The Al16 alloy shows a less negative Gibbs free energy than the Al18 alloy. As a cornerstone of metastability, the intrinsic thermodynamic instability enables the targeted deformation‐coupled transformation. Electron localization function (ELF) analysis (Figure 1h) reveals localized electron distribution around Al‐Ti, Al‐Zr, and Al‐V pairs, reflecting pronounced covalent character, while other atomic pairs exhibit metallic bonding. This mixed bonding state suggests that the strength of the alloy is largely governed by the covalent X‐Al interactions, whereas the metallic bonding facilitates plastic deformability, a structural basis for coupling dislocation slip with stress‐induced transformation.

### 2.2 Room‐Temperature Tensile Properties: Strength‐Ductility Synergy

**FIGURE 2:**
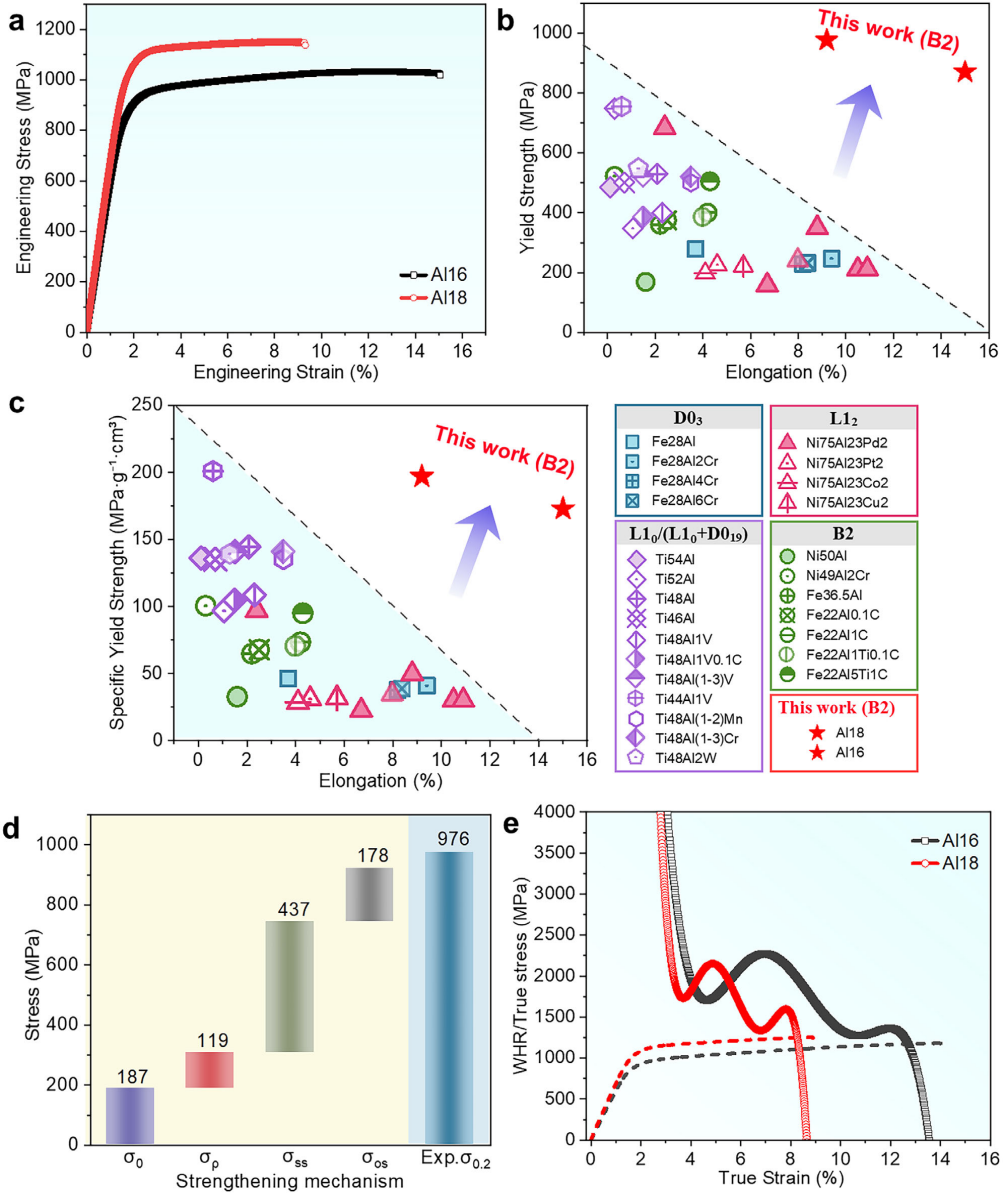
Mechanical properties of the Alx alloys and comparison with other bulk intermetallics. (a) Engineering stress–strain curves of the Alx alloys at room temperature. (b, c) YS and specific YS vs. EL of the present alloys compared with other ordered alloys in the literature [5, 6, 11, 12, 13, 42, 43, 44]. (d) The contribution of strengthening mechanisms to the YS of Al18 alloy. (e) The true stress–strain curves and WHR curves.

Figure 2a presents the representative room‐temperature tensile stress–strain curves. The Al18 alloy exhibits YS of about 976 MPa, UTS of about 1150 MPa, and EL of about 7%. The Al16 alloy achieves an EL of about 14%, double that of the Al18 alloy, with a YS reduction of only about 100 MPa (YS ∼870 MPa, UTS ∼1018 MPa). Additional performance data are given in Table S2. Fractographic analysis shows a distinct failure mode shift. In contrast to the intergranular fracture typical of brittle intermetallics, Alx alloys exhibit dimpled transgranular surfaces (Figure S4), suggesting significant plasticity before fracture. This significant ductility enhancement stems directly from the reduced phase stability achieved by lowering the Al content, which favors the transformation‐related deformation process. Figure 2b,c compares the present alloys with reported ordered intermetallics (D0_3_, L1_0_, L1_2_, B2). The Al16 and Al18 alloys deliver a specific yield strength of about 173 and 197 MPa·g^−1^·cm^3^, respectively, which are attractive for lightweight structural applications. Relative to conventional ordered intermetallics and recent high‐entropy ordered designs, the present alloys achieve a more favorable balance of strength, tensile ductility, and density.

The YS arises from a synergistic interplay of multiple strengthening mechanisms, including lattice friction stress (σ_0_), dislocation strengthening (σ_ρ_), solid solution strengthening (σ_ss_), and order strengthening via APBs (σ_os‐APB_). Given the coarse grain size (∼250 µm), GB strengthening (σ_GB_) is negligible. Quantitative analysis (Figure 2d) for Al18 alloy indicates that σ_0_ and σ_ρ_ together contribute about 33% to the YS, with the remainder dominated by σ_ss_ and σ_os‐APB_. Significant atomic size mismatch among constituent elements induces pronounced lattice distortion, which is further amplified by the multi‐element non‐stoichiometric nature of the B2 lattice, giving an estimated σ_ss_ contribution of about 430 MPa. Different from conventional intermetallics, where high APB energy (e.g., NiAl at 810 mJ/m^2^) severely restricts dislocation motion and promotes brittleness [39, 40, 41], the first‐principles calculations for Al18 alloy give a much lower APB energy of 38 mJ/m^2^. This reduced APB energy facilitates substantial order strengthening while maintaining adequate dislocation mobility necessary for cross‐slip and plastic deformation. Thus, the combination of enhanced solid solution strengthening and a dislocation‐compatible APB strengthening resolves the classic strength‐ductility conflict in intermetallics. The true stress–strain curves and work hardening rate (WHR) curves are shown in Figure 2e. Both alloys exhibit continuous work hardening over a wide true strain range, with peak WHR approaching approximately 2.3 GPa. Such strong and effective work‐hardening capability delays plastic instability and necking, enabling large uniform EL, a hallmark of the TRIP effect. In addition, even though the B2 phase is metastable and undergoes phase transformation at elevated temperatures (Figure S5), the Al18 alloy maintains excellent tensile performance, with a strength of ∼1260 MPa at 500°C and over 300 MPa at 700°C, and aging does not compromise its strength (Figure S6). It substantially outperforms TiAl and Ti64 alloys at 500°C, demonstrating its promise for short‐term high‑temperature applications.

### 2.3 Cooperative Deformation Mechanisms: Dislocation Slip and Ordered TRIP

Microstructural observations reveal that the exceptional work hardening originates from the cooperative operation of two mechanisms within the ordered matrix: (i) {110}<111> dislocation slip and (ii) a stress‑induced B2→D0_19_‐α_2_ martensitic transformation. Such ordered TRIP is distinct from conventional TRIP in disordered alloys or in NiTi/ZrCoNi alloys [45, 46, 47, 48].

#### 2.3.1 Activation of {110}<111> Dislocation

**FIGURE 3:**
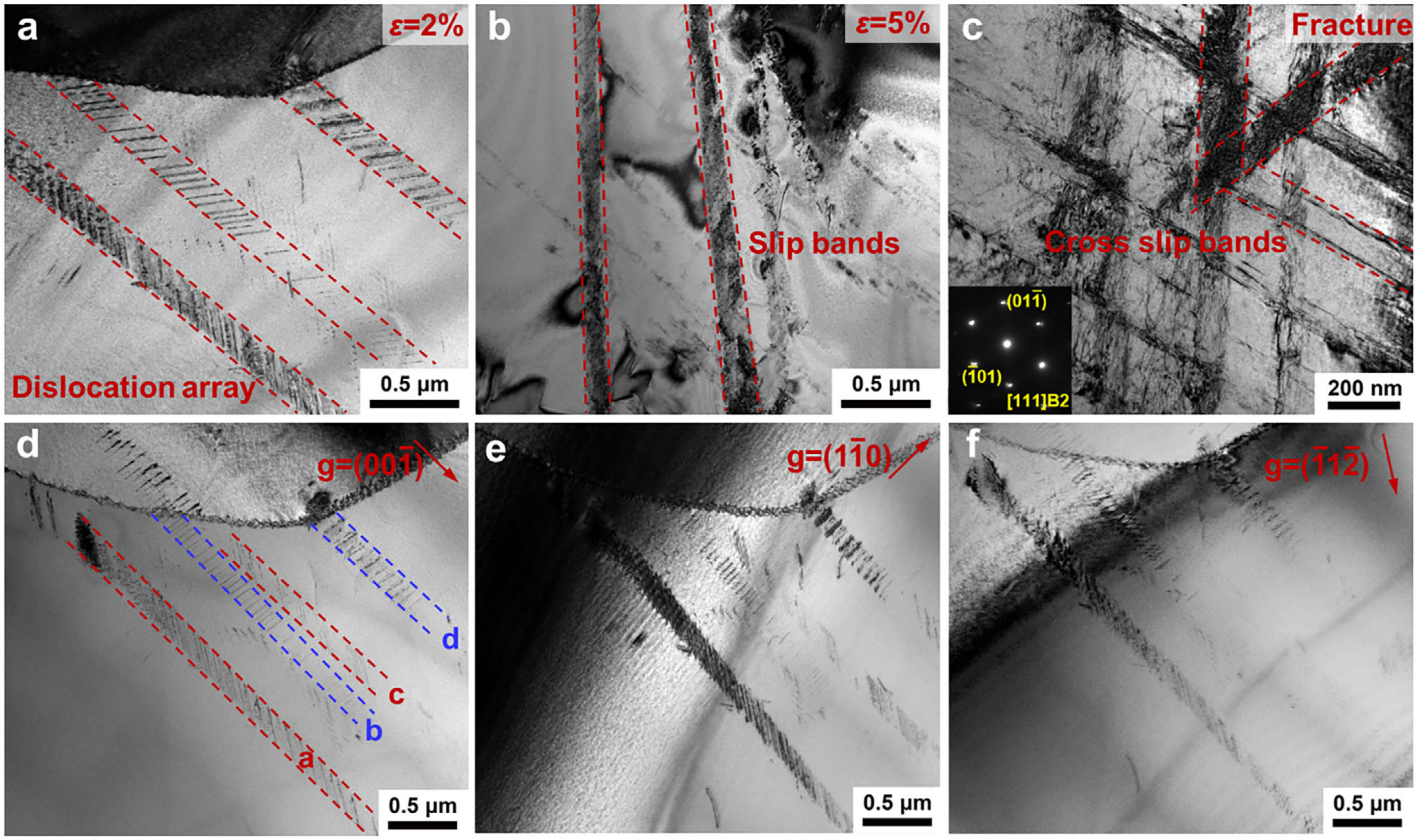
Dislocation behavior of the Al18 alloy during tensile deformation. (a–c) Dislocation morphology of Al18 alloy at 2%, 5%, and ∼8% (fracture) strains, respectively. With increasing strain, the dislocation configuration evolves from slip traces to final cross‐slip bands. (d–f) The STEM images of dislocations at a strain of 2% with different vectors ***g*** of (00‐1), (1‐10) and (‐11‐2), respectively, along with the [110] zone axis. Based on the extinction criterion ***g·b*** = 0, the visibility of dislocation traces (a–d) varies under ***g***‐vectors.

The severe GB embrittlement in intermetallics can be solved through extrinsic means such as elemental segregation or disordered interface engineering [49, 50]. Atomic‐resolution TEM shows the as‐cast Al18 alloy retains fully ordered GBs even at high misorientation angles (Figure S3c,d), indicating intrinsic GB cohesion without compositional modification. Dislocation traces emerge within grains at 2% strain and evolve into dense slip bands with increasing deformation. Extensive 60° cross‐slip is observed in the fractured Al18 alloy (Figure 3a–c), with similar dislocation accumulation in Al16 (Figure S7). Diffraction analysis reveals complex slip band networks along {110} planes, consistent with the operation of multiple active slip systems. By examining dislocation visibility under different ***g***‐vectors (Figure 3d–f and Tables S3 and S4), the Burgers vector is identified as <111>, satisfying the ***g·b*** = 0 extinction criterion. This determines {110}<111> as the operative slip system, which possesses sufficient variants to accommodate polycrystalline plasticity. Figure S7 confirms consistent <111> slip directionality in the Al16 alloy with 6% strain. However, potential activation of secondary systems remains possible [51]. It is noteworthy that dislocations can move and cross GBs during the tensile process (Figure 3d and Figure S7), indicating that GBs accommodate slip transmission and delay the formation of dislocation pile‐ups and microcrack initiation.

#### 2.3.2 Stress‐Induced B2→D0_19_‐α_2_ Ordered Martensitic Transformation

**FIGURE 4:**
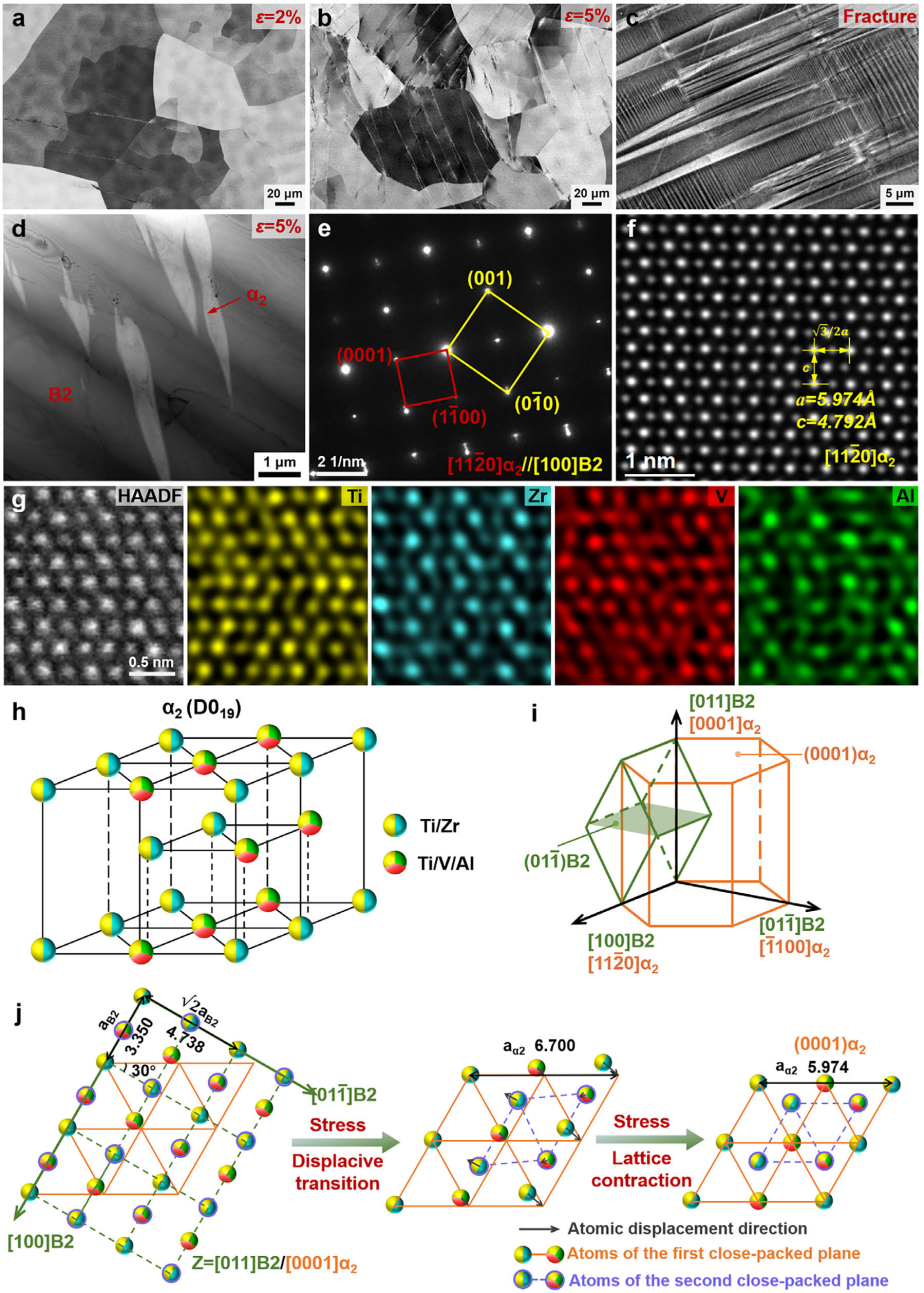
Structure of the D0_19_‐α_2_ phase of Al18 alloy. (a–c) ECCI images of martensitic phase morphology at strains of 2%, 5%, and fracture (∼8%), respectively. (d) The fractured microstructure with distinct phase characteristics (the white regions corresponding to the α_2_ phase and the grey area representing the matrix B2 phase). (e) The corresponding SAED pattern of B2 and α_2_ phases showing the orientation relationship. (f) High‐resolution HAADF‐STEM image showing the ordered structure of D0_19_ martensite taken with the [11‐20] zone axis. (g) Atomic‐resolution HAADF‐STEM images and corresponding EDX mapping along the [11‐20] zone axis showing the atomic occupancy configuration. (h) The D0_19_ superlattice structure of α_2_ martensite. (i) The schematic illustration of the orientation relationship between D0_19_‐α_2_ and B2 phases. (j) The stress‐induced displacive transition observed along the B2[110]//α_2_[0001] zone axis demonstrating the shear transformation mechanism from B2 to α_2_ phase.

Beyond dislocation slip, a novel stress‐induced martensitic transformation from the B2 matrix to a D0_19_‐α_2_ phase is identified as a central contributor to the ductility. TEM‐EDX confirms that this martensitic phase is an allotropic transformation without compositional change (Figure S8a). Post‐deformation microstructure reveals that predominant stress concentration near martensite interfaces can effectively divert stress accumulation away from GBs (Figure S8b–e), allowing dislocations to continue transmitting across GBs and keeping the local stress below the intergranular fracture threshold. Electron channeling contrast imaging (ECCI) on specimens interrupted at different deformation stages (Figure 4a–c and Figure S9) reveals that transformation initiates at 2% strain in Al18, and with increasing strain, lamellar martensite progressively penetrates entire grains and crosses GBs, forming a hierarchical structure with feature sizes from nanometers to micrometers. A similar dynamic process of the martensitic transformation is observed in Al16 alloy, as shown in Movie S1 and Figure S10. Throughout plastic deformation, the competing contributions of phase transformation and dislocation slip mechanisms, as reflected by WHR curves, dissipate strain energy through their joint action to attain optimal strength‐ductility synergy.

Unlike conventional TRIP alloys such as NiTi (B2→B19′) and ZrCoNi (B2→B33) [37, 47], the present alloys transform into a D0_19_‐α_2_ phase. The orientation relationship is identified as {0001}α_2_//{110}B2, <11‐20>α_2_//<100>B2 (Figure 4d,e and Figure S8d). Atomic‐resolution TEM along [11‐20] shows the D0_19_ structure with lattice parameters a = 5.9739 Å, c = 4.7917 Å (Figure 4f). EDX mapping indicates that Zr atoms exclusively occupy the Wyckoff position 6h; Ti, V, and Al atoms share the Wyckoff positions 6h and 2c (Figure 4g,h). Based on this atomic occupancy and orientation relationship, a transformation pathway is proposed (Figure 4i,j): tensile stress along the [110]B2 triggers migration of vertex atoms to form the [0001]α_2_ direction, while body‐centered atoms undergo displacive contraction along [001]B2. This transformation differs from classic Burgers orientation relationships, {0001}α′/α_2_ //{110}β/B2 and <11‐20>α′/α_2_ //<111>β/B2, commonly observed in Ti alloys. Thermodynamically, the D0_19_‐α_2_ phase exhibits greater stability than the B2 phase (total energies: −7.478 vs. −7.443 eV/atom), indicating a driving force for transformation. Figure S11 further confirms that D0_19_ stability increases with Zr content. In contrast, both the B19′ (−6.569 eV/atom) and B33 (−7.395 eV/atom) phases are calculated to be higher in energy than the B2 phase, making the B2→D0_19_ path energetically more favorable.

**FIGURE 5:**
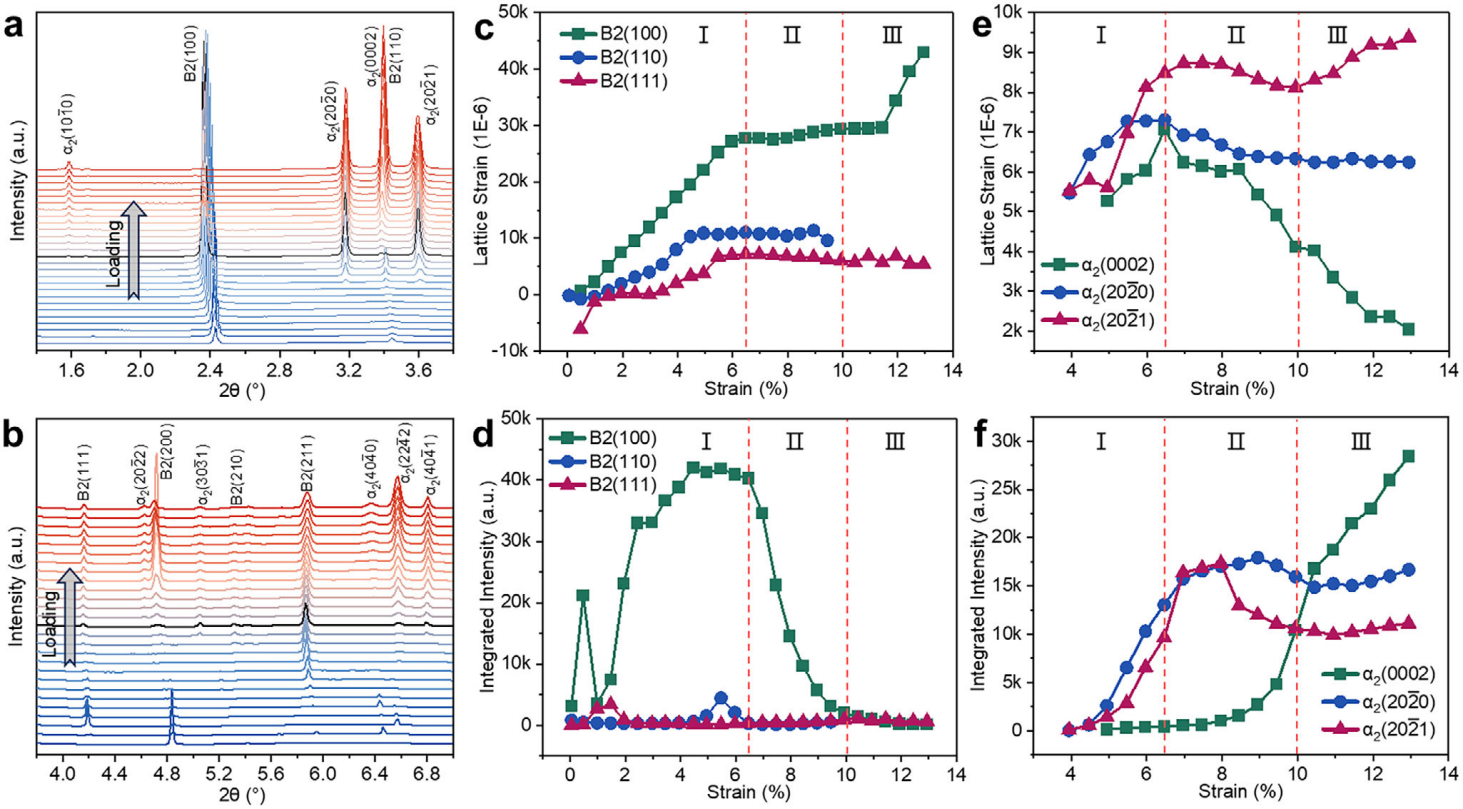
In situ synchrotron HE‐XRD for the Al16 alloy. (a, b) HE‐XRD along the loading direction. (c, e) Lattice strain as a function of strain on B2 and α_2_ phases, respectively. (d, f) Integrated intensity as a function of strain on B2 and α_2_ phases, respectively.

To directly monitor the transformation under load, in situ synchrotron high‐energy X‐ray diffraction (HE‐XRD) was performed on the Al16 alloy during tensile testing (Figure 5a,b and Figure S12). Diffraction reflections from the stress‐induced martensite emerge at a strain of 4% (∼800 MPa). The pronounced peak shifting and broadening observed during deformation, particularly at elevated strain levels, signify significant lattice distortion and the progressive accumulation of micro‐stresses. The deformation process can be divided into three stages based on lattice strain and intensity evolution (Figure 5c–f). Stage I (0%–6.4% strain) involves the elastic deformation and martensitic initiation. Lattice strains accumulate rapidly, and B2(100) intensity increases due to grain reorientation. Stage II (6.4%–10% strain) is the martensitic burst stage. Lattice strain of the B2 phase plateaus while its overall intensity declines. Most α_2_ reflections show decreased strain and intensity, but α_2_(0002) exhibits a preferential intensity increase, highlighting the orientation dependence of the transformation. Stage III (10%–14% strain) corresponds to the martensitic steady stage. The B2(110) peak eventually vanishes, while α_2_ reflections persist with increasing intensity, confirming that martensite becomes the predominant deformation mechanism at large strains. These observations are consistent with the in situ microstructural observations (Figure S10). Notably, in the Al16 alloy, nanoscale amorphous bands form during tensile necking (Figure S13), a phenomenon also reported in disordered TRIP and high‐entropy alloys [52, 53, 54, 55], further contributing to plastic accommodation. However, in the two alloys investigated here, room‐temperature stress‐induced nanoscale amorphization is observed only in the fractured Al16 alloy, driven by local strain accumulation and interface stress concentrations, and is absent in Al18 due to its lower plasticity.

### 2.4 Comparison With Conventional B2‑TRIP Systems

Conventional TRIP alloys exhibit different phase transformation behaviors. The first is the disordered TRIP effect, observed in metastable titanium alloys where the β phase (body‐centered cubic, BCC) transforms under stress into hexagonal α′ or orthorhombic α′′ martensites. These product phases are supersaturated solid solutions that retain specific orientation relationships but lack long‐range chemical order. The second is the TRIP effect from an ordered parent phase. When the BCC parent phase is chemically ordered into a B2 structure, stress‐induced ordered transformations occur. To place the present ordered TRIP effect in context, Table 1 summarizes key characteristics of representative B2‑based TRIP alloys. NiTi exhibits excellent tensile EL (>30%) but relatively low YS (<420 MPa) due to the transformation to B19′ martensite. Although ZrCoNi can achieve plasticity greater than 6% via the B33 martensitic transformation, its YS does not exceed 250 MPa. These martensitic transformations are displacive, involving only atomic shear and interplanar shuffling without long‐range diffusion. As atoms lack the opportunity to exchange sublattice sites, the chemical order is retained in the martensitic product phase.

**TABLE 1:** Comparison of B2‐based TRIP alloys.

Alloy system	Transformation	YS (MPa)	EL (%)	Product phase	Refs.
NiTi	B2 → B19′	∼200–420	>30	Monoclinic	[36, 46]
ZrCoNi	B2 → B33	∼90–250	∼2–25	Orthorhombic	[37, 38, 47]
Al16 (this work)	B2 → D0_19_(α_2_)	∼870	∼14	Hexagonal	—
Al18 (this work)	B2 → D0_19_(α_2_)	∼976	∼7	Hexagonal	—

The novelty of the present ordered transformation lies primarily in its product phase and crystallographic relationship. Through compositional design and phase stability control, the Ti‐Zr‐V‐Al alloy undergoes a stress‐induced B2→D0_19_ transformation. The resulting orientation relationship, {0001}α_2_//{110}B2 and <11‐20>α_2_//<100>B2, differs from those typical of conventional disordered transformations in titanium alloys. The Al16 and Al18 alloys achieve much higher yield strengths (∼870 and ∼976 MPa) while maintaining favorable elongations (∼14% and ∼7%), and the transformation product (D0_19_‐α_2_) is itself an ordered intermetallic with high strength. Both parent and product phases in our alloy retain long‑range order, offering the potential for higher intrinsic strength and thermal stability. This comparison underscores the distinct position of the present alloys: they demonstrate that ordered TRIP can overcome the strength‐ductility trade‐off in B2 aluminides by coupling a moderate transformation strain with high lattice resistance and low APB energy.

## 3 Conclusion

In summary, controlled reduction of B2 phase stability in Ti‐Zr‐V‐Al medium‐entropy ordered alloys enables the cooperative activation of {110}<111> dislocation slip and a stress‑induced B2→D0_19_‐α_2_ ordered martensitic transformation. The latter represents an atypical transformation pathway distinct from both the classical Burgers relationship and the B2→B19′/B33 transformations known in other systems. This ordered TRIP effect achieves a tensile EL of about 7%, together with YS of ∼976 MPa, UTS of about 1.1 GPa, and specific yield strength of about 197 MPa·g^−1^·cm^3^, a combination that surpasses most lightweight B2 intermetallics. These findings establish metastability engineering as an effective strategy for improving the tensile deformability of ordered intermetallic alloys without compromising their strength advantage, and provide guidance for designing high‐strength ordered alloys for lightweight structural applications.

## 4 Experimental Section

### 4.1 Alloys Preparation

Polycrystalline Ti_50_Zr_46‐x_V_4_Al_x_ (at.%; x = 16, 18) ingots were fabricated via arc melting under an argon atmosphere. The purity of all alloying elements exceeded 99.99 wt.%. To mitigate aluminum volatilization, the proportion of Al was set at 1.03 times the nominal composition. The alloys were remelted 5–6 times to ensure compositional uniformity of the alloy. The ingots were cast into a water‐cooled copper mold with cross‐sectional dimensions of 10 mm × 10 mm × 55 mm.

### 4.2 Microstructure Characterization

The phase structures of the alloys were detected using XRD (Rigaku Smart Lab 9 kW) with Cu Kα radiation (wavelength of 1.5406 Å) in a 2θ range from 20° to 100° at a scanning rate of 10°/min.

The grain size, orientation, phase transformation behavior, and stress distribution of these alloys were analyzed using a ZEISS SUPRA 55 scanning electron microscope (SEM) equipped with an EBSD detector, and a Tescan MIRA 3 XMH SEM fitted with a coaxial transmission Kikuchi diffraction detector (Bruker OPTIMUS). The phase transformation behavior of the alloy under different strain conditions was investigated using a SEM (ZEISS SUPRA 55) equipped with an ECCI attachment, which was operated at an accelerating voltage of 20 kV, a beam current of 2 nA, and a working distance of 6 mm. Images were acquired using a 6‐quadrant in‐lens backscatter detector without tilting the sample stage. All such samples were subjected to precision grinding followed by electrolytic polishing, which consisted of 65 vol% methanol, 30 vol% nitric acid, and 5 vol% ethylene glycol monobutyl ether at a voltage of 25 V, a temperature below −25°C, and a duration of 60 s.

A FEI Tecnai G2 F20 field‐emission TEM operated at 200 kV and equipped with a Bruker EDX detector was employed to characterize the phase structure, microstructure morphology, element distribution, and dislocation configuration of as‐cast and deformed samples. Atomic‐resolution structures and chemical distributions of different phases were co‐analyzed using spherical aberration‐corrected TEM (FEI Themis Z) equipped with a Super‐X EDX under imaging modes of STEM and HAADF. Samples were prepared by ion thinning or electrolytic double‐jet polishing to meet the requirements for TEM observation. The solution consisted of 88 vol.% methanol, 8 vol.% perchloric acid, and 4 vol.% glacial acetic acid, with the polishing temperature range of −30°C to −25°C and a voltage of 25 V.

The compositional uniformity of the matrix was precisely analyzed using an APT (LEAP 4000X SI). The measurements were conducted at a temperature of 50 K with a pulse frequency of 200 kHz and a pulsed ultraviolet laser energy of 50 pJ. The APT tips were prepared by focused ion beam and welded onto Cu holders or Si pillar arrays.

### 4.3 Tensile Testing

The room‐temperature tensile properties of the alloys were tested at a strain rate of 1 × 10^−3^ s^−1^ on a universal electronic testing machine (CMT4105) equipped with a mechanical extensometer (class 0.5) with a gauge length of 15 mm. The tensile specimens were machined into a dog‐bone shape with the gauge section dimensions of 2 mm × 3 mm × 18 mm. Before the tensile tests, the surfaces of the specimens were finely ground with 2000# sandpaper to remove the marks and grooves left by processing. High‐temperature tensile tests were conducted on a Wance TSE105D dual‐column electronic universal testing machine with a strain rate of 1 × 10^−3^ s^−1^ and a gauge length of 6 mm.

### 4.4 In Situ Synchrotron High‐Energy X‐ray Diffraction

In situ HE‐XRD tensile experiments were performed at the PETRA III P07B beamline of Deutsches Elektronen‐Synchrotron (DESY), Germany. A monochromatic beam with an energy of 87.109 keV (wavelength 0.14235  Å) was focused to a spot size of 0.7  mm  ×  0.7  mm. A transmission geometry was employed, and the resulting Debye‐Scherrer rings were recorded by a two‐dimensional area detector. Instrument calibration was conducted using a LaB_6_ standard to determine the sample‐to‐detector distance and other necessary parameters. Dog‐bone‐shaped specimens with a gauge section of 20   ×  4.5   ×  1  mm^3^ were deformed at a strain rate of 1  ×  10^−3^ s^−1^. The collected two‐dimensional patterns were azimuthally integrated along the loading direction within an angular range of 90°  ±  5° to obtain one‐dimensional diffraction profiles.

### 4.5 First‐Principles Calculations

To evaluate the phase stability of the metastable ordered B2 matrix and ordered martensitic α_2_ phase in the Ti‐Zr‐V‐Al alloys, first‐principles calculations were employed to predict the formation energy by constructing supercell structures to simulate chemical site occupations. The B2 phase supercell contained 100 atoms. The possible atomic ordered occupations were simulated mainly by constructing the occupation probability of Zr atoms at the vertex positions, and the magnitude of the structural ordering energy determined the phase stability. Therefore, nine models were constructed, including one disordered model and eight ordered occupation models. Herein, the ordering energy refers to the energy difference between the ordered structure where alloying elements are distributed in specific sublattices and the disordered structure with the same composition in a completely random configuration. Since the ordered martensitic D0_19_‐α_2_ phase is transformed from the B2 phase, the supercell model with fixed atomic occupations (containing 96 atoms) can be directly constructed according to the orientation relationship to calculate the phase formation energy. To further assess the influence of Zr content on the phase stability of the D0_19_ structure, two additional models (Ti_78_V_4_Al_18_ and Ti_64_Zr_14_V_4_Al_18_, at.%) were constructed. In addition, to quantitatively evaluate the APB energy, a non‐conservative APB model with 100 atoms on the (100) plane was constructed, featuring an APB area of 6.7002 Å × 16.7505 Å. The plane‐wave cutoff energy was set to 520 eV, and the energy convergence threshold was 10^−5^ eV.

## Author Contributions

**Chaohua Li**: writing – original draft, writing – review and editing, validation, formal analysis, data curation, methodology, investigation, conceptualization. **Yidong Wu**: writing – review and editing, visualization, methodology, investigation, funding acquisition, conceptualization. **Boyuan Zheng**: methodology, investigation, visualization. **Xu Zhang**: formal analysis, methodology, validation. **Xiongjun Liu**: conceptualization, writing – review and editing, supervision. **Wenli Song**: methodology, supervision, validation, writing – review and editing. **Ning An**: investigation, project administration, formal analysis. **Chengbo Xiao**: supervision, project administration, methodology. **Xidong Hui**: funding acquisition, project administration, supervision, writing – review and editing, formal analysis, conceptualization, resources.

## Funding

This study was financially supported by the National Natural Science Foundation of China (grants U22A20105 and 52373278).

## Conflicts of Interest

The authors declare no conflicts of interest.

## Supporting information




**Supporting File 1**: advs77739‐sup‐0001‐SuppMat.docx.

**Supporting File 2**: advs77739‐sup‐0002‐MovieS1.mp4.

## Data Availability

The data that support the findings of this study are available from the corresponding author upon reasonable request.
