# Supplementary material for: A New Ordered Transformation‐Induced Plasticity Enables Exceptional Strength and Ductility in a B2 Medium‐Entropy Alloy
Source: Adv Sci (Weinh). 2026 Sep 16:e77739. Online ahead of print. doi: 10.1002/advs.77739 (PMC13583113; doi:10.1002/advs.77739)
Supplement: Supplementary file 1 [file ADVS-9999-e77739-s002.docx]

**Supporting Information for**

A new ordered transformation-induced plasticity enables exceptional strength and ductility in a B2 medium-entropy alloy

Chaohua Li, Yidong Wu, Boyuan Zheng, Xu Zhang, Xiongjun Liu*, Wenli Song*, Ning An, Chengbo Xiao, Xidong Hui*

C. Li, Y. Wu, B. Zheng, X. Zhang, X. L, X. Hui

State Key Laboratory for Advanced Metals and Materials, University of Science and Technology Beijing, Beijing 100083, China

W. Song

Institute of High Energy Physics, Chinese Academy of Sciences, 100049, Beijing, China

Spallation Neutron Source Science Center, 523803, Dongguan, China

N. An

Institute of Materials Research, Beiiing Beiye Advanced Materials Co., Ltd, Beijing, 100192, China

C. Xiao

Science and Technology on Advanced High Temperature Structural Materials Laboratory, Beijing Institute of Aeronautical Materials, Beijing, 100095, China

C. Li, Y. Wu

These authors contributed equally to this work.

**Email:** xdhui@ustb.edu.cn; xjliu@ustb.edu.cn; songwl@ihep.ac.cn

**Calculations of yield strength contributions**

The tensile yield strength contributions of Al18 alloy at room temperature can be expressed as

$\sigma_{y}=\sigma_{0}+\sigma_{\rho}+\sigma_{ss}+\sigma_{os}$	(S1)

where σ_0_ is the lattice friction stress, σ*_ρ_* is the dislocation strengthening, σ*_ss_* is the solid solution strengthening, and σ*_os_* is the order strengthening via antiphase boundaries. Here, the grain boundary strengthening can be neglected due to the large grain size.

The lattice friction stress σ_0_ refers to the stress corresponding to the inherent resistance encountered when dislocations move in the lattice, and it can be calculated using the mixing principle of multiple elements^[1]^.

$\sigma_{0}=\sum_{i=1}^{n}{c_{i}\sigma}_{i}$	(S2)

where *c_i_* and *σ_i_* represent the atomic percentage and basic strength of element *i*. The basic strengths of Ti, Zr, V, and Al are 195 MPa, 280 MPa, 150 MPa and 30 MPa, respectively.

The dislocation strengthening *σ_ρ_* can be calculated using the Taylor model^[2]^, expressed as

$\sigma_{\rho}=\alpha MGb\sqrt{\rho}$	(S3)

where *α* is a constant related to dislocation interaction (0.4), *M* is the Taylor factor (3.067)^[3]^, *G* is the shear modulus (23 GPa), *b* is the Burger's vector ($\sqrt{3}a/2$, *a* is the lattice constant, the density of dislocation can be assessed as *ρ*=2*KAM_ave_*/*μb*^[4]^. Here, *KAM_ave_* is the average localized misorientation measured by EBSD (0.243°), *μ* is the unit length (8 μm).

The solid solution strengthening σ*_ss_* can be evaluated according to the modified Labush model by

$\sigma_{ss}=\frac{G}{45}{(\Sigma_{i}\varepsilon_{i}^{2}c_{i})}^{2/3}$	(S4)

where *ε_i_* is a mismatch parameter caused by solute elements. Further calculations can refer to relevant literature^[5]^.

The order strengthening^[6]^is given by:

$\sigma_{os}=0.81M\frac{\gamma_{APB}}{2b}{(\frac{3\pi f}{8})}^{1/2}$	(S5)

where *γ_APB_* is the antiphase boundary energy following lattice distortion obtained through first principles calculations (38 mJ/m²), *f* is the volume fraction of B2 phase (*f* =1).

**Figure S1. Structure and morphology of as-cast Alx alloys.** (a) Phase structure of as-cast Alx alloys. (b) Inverse pole figure (IPF) of Al16 alloy revealing the polycrystalline morphology and grain size.

**Figure S2. The elemental distribution differences between matrix and APDs in Al18 alloy.** The alloy exhibits a homogeneous elemental distribution.

**Figure S3. TEM images and crystal structure of as-cast Al16 alloy**. (a) The morphology of the matrix with APD. The corresponding SAED pattern of [100] zone axis proves the ordered structure in Al16 alloy. (b) High-resolution HAADF-STEM image of Al16 alloy along [100] zone axis with a lattice parameter of 3.384 Å. (c) The macroscopic morphology of the grain boundary between grains with misorientation. (d) HAADF-STEM image of atomic-resolution grain boundary, where the interface maintains completely ordered structure.

**Figure S4. Fracture morphology of Alx alloys**. (a1-a2) The fracture characteristics of Al18 alloy consist of cleavage planes, river patterns, and dimples. (b1-b2) The fracture behavior of Al16 alloy is dominated by dimple and tear ridges, which displays improved ductility compared to Al18 alloy.

**Figure S5. Microstructures and phase structure of the Al18 alloy after aging at different temperatures and for various durations**. (a1) 500℃-30 min. (a2) 500℃-24 h. (b1) 700℃-30 min. (b2) 700℃-24 h. (c1) 900℃-30 min. (c2) 900℃-24 h. (d) XRD pattern of Al18 alloy after aging at 700 °C for 2 h.

 **Figure S6. High‑temperature tensile properties of Al18 alloy under various conditions.** (a) Elevated‑temperature tensile properties of the alloy in the as‑cast and aged conditions over the temperature range of 500–700 °C. (b) Comparison of high‑temperature properties with typical intermetallics and titanium alloys^[7–10]^.

**Figure S7. Dislocation evolution behavior of Al16 alloy during tensile deformation**. (a) Dislocation slip traces at a strain of 2%. (b) The formation of slip bands at a strain of 6%. (c) Entanglement and proliferation of dislocations in the fractured alloy (strain of 14%). (d-f) Dislocations morphology at a strain of 6% under the **g** = (00) along [100], **g** = (01) along [100] and **g** = (12) along [110], respectively.

**Figure S8. EDS and TKD-EBSD images of deformed Al18 alloy corresponding to Figure 4d**. (a) The elemental distribution differences between parent phase B2 and martensite α_2_ phase. (b) Band contrast image. (c) Phase distribution mapping. (d) IPF mapping. (e) KAM mapping.

**Figure S9. ECCI images of the phase transformation process for Al16 alloy with different strains**. (a) Initial phase transformation at a strain of 2%. (b) Phase transformation morphology during mid-stage deformation (6%). (c) Post-fracture phase transformation microstructure (~14%).

**Figure S10.** **Microstructural evolution of the Al16 alloy during in-situ tensile deformation**.

**Figure S11.** **The ordering energy of D0_19_ structure for Ti_78-y_Zr_y_V_4_Al_18_ (at.%, y = 0, 14, 28, referred to as Zry alloy) alloys**. The phase stability of the D0_19_ structure shows a positive correlation with Zr content in this alloy system.

**Figure S12.** **In situ synchrotron tensile stress-strain curve of the Al16 alloy**.

**Figure S13.** Formation of amorphous bands in deformed Al16 alloy. (a) Bright TEM image of numerous amorphous bands. (b) Enlarged dark TEM image of the amorphous band. (c) The corresponding crystal plane of amorphous bands along [110] B2 zone axis. (d) The interface between the amorphous region and the ordered B2 matrix. (e) The enlarged amorphous region from (d). (f) The enlarged ordered atomic arrangement along [111] B2 zone axis from (d).

**Table S1** The calculated values of empirical parameters for Alx alloys.

| No. | *△S_mix_*(J/(K·mol)) | △Hmix(kJ/mol) | *δ*(%) | *VEC* | *Ω* | *Λ* |
| --- | --- | --- | --- | --- | --- | --- |
| Al18 | 9.48 | -20.470 | 4.87 | 3.86 | 0.8436 | 0.40 |
| Al16 | 9.39 | -18.810 | 4.94 | 3.88 | 0.9213 | 0.38 |

**Table S2** Room-temperature tensile properties (mean ± SD) of the Al16 and Al18 alloys.

| Alloy | UTS/MPa | YS/MPa | EL/% |
| --- | --- | --- | --- |
| Al16 | 1025 ± 6 | 839 ± 23 | 11.7 ± 2.2 |
| Al18 | 1075 ± 54 | 917 ± 42 | 7.2 ± 0.7 |

**Table S3** The *g•b*=0 extinction condition analysis of the dislocations for Al18 alloy marked in Figure 3. The letters “V” and “I” represent “visible” and “invisible” respectively.

| Zone axis | **g** | Dislocations | | | |
| --- | --- | --- | --- | --- | --- |
|  |  | a | b | c | d |
| [110] | (00) | V | V | V | V |
| [110] | (10) | V | V | V | V |
| [110] | (1) | V | V | I | V |
| Possible Burgers vector | | [11] or [1] | | | |

**Table S4** The ***g****•****b***=0 extinction condition analysis of the dislocations for Al16 alloy marked in Figs. 7d–f. The letters “V” and “I” represent “visible” and “invisible” respectively.

| Zone axis | **g** | Dislocations |
| --- | --- | --- |
| [100] | (00) | V |
| [100] | (01) | V |
| [110] | (12) | I |
| Possible Burgers vector | | [11] or [1] |

**References**

1. Z. H. Guo, X. Y. Shen, F. Liu, et al., “Microstructure and mechanical properties of Alx(TiZrTa_0.7_NbMo) refractory high-entropy alloys,” *Journal of Alloys and Compounds* 960 (2023): 170739.
2. R. K. Nutor, Q. P. Cao, R. Wei, et al., “A dual-phase alloy with ultrahigh strength-ductility synergy over a wide temperature range,” *Science Advance* 7 (2021): 4404.
3. S. Han, S. Laube, Z. H. Chen, et al., “Operative slip system and dislocation behavior in a brittle refractory high-entropy alloy with the B2 ordered structure,” *Journal of Alloys and Compounds* 1020 (2025): 179361.
4. R. Huang, L. K. Zhang, A. Amar, et al., “Achieving excellent uniform tensile ductility and strength in dislocation-cell-structured high-entropy alloys,” *International Journal of Plasticity* 181 (2024): 104079.
5. R. Labusch, “A statistical theory of solid solution hardening,” *Physica Status Solidi* 41 (1970): 659–669.
6. D. N. Seidman, E. A. Marquis, D. C. Dunand, “Precipitation strengthening at ambient and elevated temperatures of heat-treatable Al (Sc) alloys,” *Acta Materialia* 50 (2002): 4021–4035.
7. Y. Kaneno, K. Asao, M. Yoshida, et al., “Tensile properties of recrystallized B2 CoZr intermetallic alloys,” *Journal of Alloys and Compounds* 456 (2008) 125–134.
8. Y. Kaneno, T. Takasugi, S. Hanada, “Tensile property and fracture behavior of hot-rolled CoTi intermetallic compound,” *Materials Science and Engineering A* 302 (2001) 215–221.
9. K. Hashimoto, “High-temperature tensile properties of Ti-Al-X (X = Cr, W) consisting of α_2_, β and γ in three phases,” *Materials Science Forum* 706–709 (2012) 1066–1070.
10. W. Shao, D. Peiran, J. Qiu, et al., “Tensile deformation behavior of Ti-6Al-4V sheet at elevated temperature,” *Materials Research Express* 6 (2019) 116585.
